# How to Conduct Multimethod Field Studies in the Operating Room: The iPad Combined With a Survey App as a Valid and Reliable Data Collection Tool

**DOI:** 10.2196/resprot.4713

**Published:** 2016-01-05

**Authors:** David W Tscholl, Mona Weiss, Donat R Spahn, Christoph B Noethiger

**Affiliations:** ^1^ Institute for Anesthesiology University and University Hospital Zurich Zurich Switzerland; ^2^ Leonard N. Stern School of Business New York University New York, NY United States

**Keywords:** data collection, empirical research, observation, computers, informatics, anesthesiology

## Abstract

**Background:**

Tablet computers such as the Apple iPad are progressively replacing traditional paper-and-pencil-based data collection. We combined the iPad with the ready-to-use survey software, iSurvey (from Harvestyourdata), to create a straightforward tool for data collection during the Anesthesia Pre-Induction Checklist (APIC) study, a hospital-wide multimethod intervention study involving observation of team performance and team member surveys in the operating room (OR).

**Objective:**

We aimed to provide an analysis of the factors that led to the use of the iPad- and iSurvey-based tool for data collection, illustrate our experiences with the use of this data collection tool, and report the results of an expert survey about user experience with this tool.

**Methods:**

We used an iPad- and iSurvey-based tool to observe anesthesia inductions conducted by 205 teams (N=557 team members) in the OR. In Phase 1, expert raters used the iPad- and iSurvey-based tool to rate team performance during anesthesia inductions, and anesthesia team members were asked to indicate their perceptions after the inductions. In Phase 2, we surveyed the expert raters about their perceptions regarding the use of the iPad- and iSurvey-based tool to observe, rate, and survey teams in the ORs.

**Results:**

The results of Phase 1 showed that training data collectors on the iPad- and iSurvey-based data collection tool was effortless and there were no serious problems during data collection, upload, download, and export. Interrater agreement of the combined data collection tool was found to be very high for the team observations (median Fleiss’ kappa=0.88, 95% CI 0.78-1.00). The results of the follow-up expert rater survey (Phase 2) showed that the raters did not prefer a paper-and-pencil-based data collection method they had used during other earlier studies over the iPad- and iSurvey-based tool (median response 1, IQR 1-1; 1=do not agree, 2=somewhat disagree, 3=neutral, 4=somewhat agree, 5=fully agree). They found the iPad (median 5, IQR 4.5-5) and iSurvey (median 4, IQR 4-5) to be working flawlessly and easy to use (median 5, IQR 4-5). Expert ratings also showed that the anesthesia team members (ie, the surveyed doctors and nurses) who used the iPad- and iSurvey-based tool in the OR liked it (median 4, IQR 3-4.5).

**Conclusions:**

The combination of the iPad and iSurvey provides an efficient and unobtrusive method to observe teams in their natural environment in the OR and to survey team members immediately after completing their task (ie, anesthesia induction). The expert raters positively evaluated the use of the device and user perceptions. Considering these comprehensive results, we can recommend the use of the iPad- and iSurvey-based tool for studying team performance and team member perceptions in the OR.

## Introduction

The use of computer-based data collection tools has increased rapidly over the past decade, and these tools are progressively replacing paper-and-pencil-based methods [1,2].

The Apple iPad has become a revolutionary device in terms of ease of use, versatility, and availability. Useful applications in clinical practice [3-7] and medical education [8-12] have been reported. However, evidence is lacking on whether the iPad may also provide a suitable data collection tool to assess teamwork and team performance in the operating room (OR). This is problematic as a better understanding of teamwork and performance in the OR is critical for patient safety [13]. Although there are some studies that have investigated teamwork in the OR or conducted field-based experiments with health care teams [14-17], no study has outlined the specific methodological requirements that are necessary to conduct such studies. This may limit methodological knowledge and thus impede the growth of further research in this area.

We used the iPad loaded with the iSurvey app for data collection during an extensive intervention study in the OR that included data from multiple sources. Specifically, we used the combination of the iPad and iSurvey to conduct behavioral observations of teamwork and performance during systematic observations of anesthesia inductions and to assess team member perceptions regarding key teamwork aspects (eg, information exchange, knowledge of critical information, perception of safety). These assessments involved a large number of questions, which would have made a paper-and-pencil-based approach cumbersome and error-prone, considering that we collected data in various OR areas of an academic hospital [18]. Another key challenge was the fact that we aimed to investigate anesthesia teams before, during, and after anesthesia induction. This required the use of an unobtrusive and yet reliable method to assess data [19].

In this paper, we aim to provide a detailed analysis of the factors that led to the use of the of iPad and iSurvey as our combined data collection tool, illustrate our experiences with the use of this data collection tool during the intervention study, and report the results of a follow-up study conducted with the expert raters who used the tool. With the results of this research, we contribute further methodological insights on how to conduct multimethod field studies in the OR.

## Methods

The following sections describe two study phases. In Phase 1, we used the iPad and iSurvey for a hospital-wide intervention study in which we evaluated a newly developed anesthesia pre-induction checklist (APIC study [18]). In Phase 2, we conducted a follow-up study to assess expert raters’ perceptions and experiences of using the iPad and iSurvey as a data collection tool in the OR.

### Phase 1: The APIC Study

In the APIC study [18], we tested whether the iPad loaded with iSurvey software would be a suitable tool to assess team performance and team member perceptions in the OR. Findings are based on an intervention study that tested the effectiveness of an Anesthesia Pre-Induction Checklist (APIC) using a control group design. We introduced the APIC to provide a check and briefing of safety-critical items immediately before the induction of anesthesia. The key aims of the checklist are to avoid omission errors and to improve situation awareness by promoting a shared mental model between all members of the anesthesia team.

The APIC study featured a multimethod approach comprising (1) onsite systematic observations of anesthesia inductions and (2) surveys of the observed anesthesia team members conducted immediately after the onsite observations.

We compared data from teams who used the APIC (intervention group) during anesthesia induction with teams who did not use the APIC (control group). Specifically, we tested the effects of the APIC on communication and technical performance of anesthesia teams and team members’ awareness of critical information, perceptions of safety, and perceptions of teamwork. Ethics approval was given by the ethics committee of the Canton of Zurich (KEK StV-Nr. 07/12), Zurich, Switzerland.

#### Participants

We observed a total of 205 anesthesia inductions in seven OR areas at the University Hospital Zurich, Zurich, Switzerland. We observed 105 teams (including a total of 285 team members, ie, doctors and nurses) before, and 100 teams (272 team members) after the introduction of the APIC.

#### Procedure and Measures

In the following section, we will outline (1) factors that led to the decision to use an electronic data collection tool, (2) requirements that our desired data collection tool needed to fulfill, (3) factors that led to the use of the iSurvey software specifically, (4) how we created the iPad- and iSurvey-based data collection tool, and (5) how we applied the iPad- and iSurvey-based tool during the APIC study.

##### Reasons to Use an Electronic Data Collection Tool

The decision to use an electronic data collection tool was based on the following considerations. First, we planned to observe anesthesia inductions in seven different operating areas situated in multiple locations of an academic hospital. Second, the study required large numbers of observations and involved an extensive data collection protocol (more than 60 items per observation). Third, the anesthesia teams were observed during and surveyed immediately after the anesthesia induction, which required us to use a fast and unobtrusive way to assess data.

A paper-and-pencil–based data collection method would have required the multiple data collectors to handle and keep track of large amounts of paper. We reasoned that this would have made data collection, storage, and management more time and energy consuming and more prone to errors when compared to an electronic data collection method. We thus sought a simple and reliable electronic method to collect our data.

##### Requirements for the Desired Survey Software

Before deciding on a specific survey app to be used during our research project, we defined some criteria that we considered important. As wireless Internet access could not be guaranteed in all positions inside the operating areas, we required an app that provided offline data collection. Moreover, the creation of surveys had to be easy and straightforward, without the requirement of software-programming skills. The software had to be ready for data entry within a couple of seconds, and if the data collection was interrupted during an observation, the survey had to restart at the same position after pushing the start button. We also needed to be able to use a branching logic—mandatory questions that inhibit the continuation of the survey until a question has been answered and group questions to avoid switching between survey screens in order to minimize cognitive effort of the data collectors. Also, the answers had to remain saved when going back and forth between survey screens. Finally, the app also had to have a reasonable price to fit our research budget.

##### Reasons to Use iSurvey Software

While planning the study in February 2012, before selecting a survey app, we downloaded and evaluated all survey apps that offered a free initial download on the US and Swiss Apple App Stores, using the search terms “survey” and “data collection,” in order to identify the app that best met our previously defined requirements. We evaluated the following apps: SurveyPocket by Jeremy Przasnyski (surveyanalytics site), Polldaddy by Auttomatic, Inc. (polldaddy site), iFormbuilder by Zerion Software, Inc. (iformbuilder site), and iSurvey (Harvestyourdata, Wellington, New Zealand). We also evaluated SurveyMonkey and Qualtrics, but these providers did not offer a solution that worked offline on an iPad. We decided to use iSurvey because it was the only one of these apps that saved answers when going back and forth between screens and allowed grouping of multiple answers on a single screen.

##### Creation of the Data Collection Tool

Once we made the decision to use iSurvey as the software for our data collection tool, we created the survey containing all the data collection protocol questions for the study in the password-protected user area of iSurvey site. The exact number of questions asked per observation varied because we used the iSurvey function to choose a branching logic. Using this feature, the survey directs the user to a prespecified question or information screen depending on how a question is answered. Also, control questions were included to verify that an observation was within the predefined study inclusion criteria. For example, if the question “Is this an emergency situation?” was answered with “yes,” the survey was terminated because only anesthesia inductions for elective surgery, and not emergency procedures, were to be included. We also used the function of iSurvey to randomize the order of the answers to a question to minimize common survey response biases such as the tendency to respond in the same direction on a series of questions regardless of the content.

##### Application of the Data Collection Tool During the APIC Study

We recruited 5 attending anesthesiologists (ie, each with more than 5 years of clinical anesthesia experience) to serve as expert raters of team performance and as data collectors for the survey of team member perceptions. Prior to the observations in the ORs, we conducted a training session. This session served to (1) explain the study procedure, (2) familiarize the expert raters with the data collection tool, (3) train the raters in observational skills, and (4) test the interrater reliability of the data collection tool. We explained to the expert raters how to start the iPad and iSurvey and how to upload data after an observation. We also conducted a rating of a videotaped anesthesia induction scenario together with the expert raters. To assess interrater agreement, we recorded three multi-angle videos of anesthesia induction scenarios showing different levels of team performance in a full-scale anesthesia simulator. All 5 expert raters then independently watched and rated the videos using the data collection tool, and Fleiss’ kappa was calculated.

During the data collection phase of the study, we conducted three meetings with all expert raters to address questions pertaining to the iPad- and iSurvey-based data collection tool. During the study, the expert raters answered general questions and questions about team performance (ie, communication and clinical performance). For example, a general question was “Which team member read the checklist? Consultant/resident/nurse.” An example of a team performance question was “Did the team talk about the patient allergies? Yes/no.”

The expert raters completed nominal scale-level questions (yes/no, and different choices, multiple-choice, and single best answer; for example (different choices single best answer), “Name of the OR area the observation is taking place in. OR area #1, OR area #2, etc”.

After the observation, the expert raters handed the iPad to each observed team member, who then individually and privately answered a short survey. The individual team members answered general questions and questions about their perceptions during the induction. For example, a general question was “My anesthesia experience in years? >1, 1-5, 5-10, >10 years,” or a question about team member perceptions was “How safe did I feel during this induction?” answered on a continuous Likert-type rating scale from 0% (very unsafe) to 100% (very safe). The anesthesia team members completed nominal scale-level questions, different choices (multiple-choice and single best answer), and interval scale-level questions (continuous rating scales).

The observation and team member survey procedure for anesthesia teams in both the APIC group and the control group did not differ, and teams in both groups were observed and surveyed equally by the same expert raters. These expert raters did not participate in the anesthesia inductions they observed or in the team member surveys after the inductions. Their sole purpose was to rate the anesthesia induction and administer the team member survey to the observed team members after the induction.

**Figure 1:**
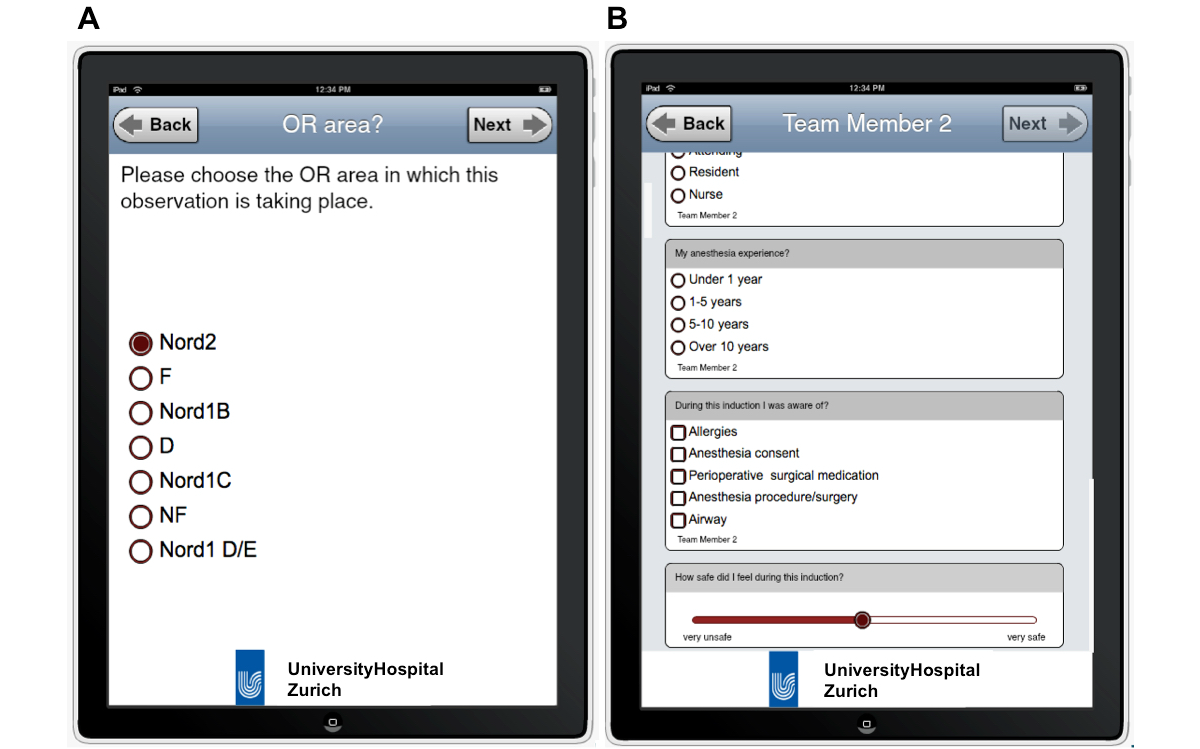
Screenshots of the iPad- and iSurvey-based data collection tool. The left shows the tool asking the data collector to name the operating area in which the observation is taking place. The right shows example questions asked during the team member survey.

**Figure 2:**
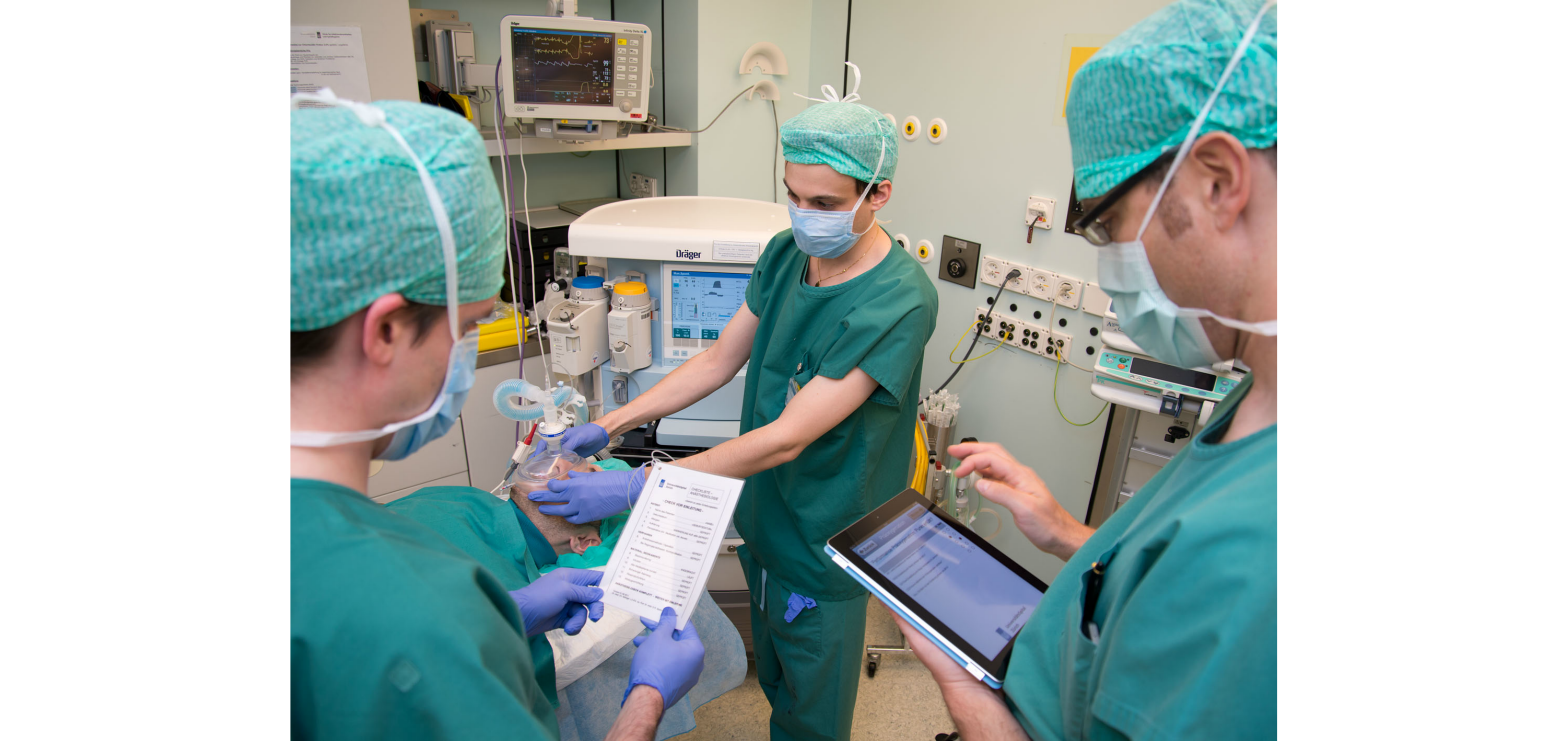
A data collector using the iPad- and iSurvey-based data collection tool to rate anesthesia team performance during a systematic onsite observation of team performance.

The collected data were downloaded from the password-protected user area from the iSurveysoft site as a MS Excel readable CSV (comma separated value) file for analysis. Figure 1 shows screenshots of the data collection tool used in this study. Figure 2 shows an example of how the data collection was conducted in the ORs.

### Phase 2: Expert Rater Survey

After the completion of the APIC study, we conducted a follow-up survey of the data collectors to assess their perceptions about the iPad- and iSurvey-based data collection tool. We aimed to receive a more in-depth understanding of the use of the iPad- and iSurvey-based tool and to assess the expert raters’ impressions of the use of the tool.

#### Participants

We conducted a survey with the same 5 expert raters who used the iPad- and iSurvey-based tool in the ORs during the observations and team member surveys of the APIC study.

#### Procedures and Measures

An email containing a single-page MS Word document as an attachment was sent to the 5 expert raters. The survey contained a total of 7 questions: 6 questions with Likert-type rating options (1=do not agree, 2=somewhat disagree, 3=neutral, 4=somewhat agree, 5=fully agree) and one open-ended question, which enabled the expert raters to provide any kind of feedback. Table 1 contains all questions (1-7) as well as the raters’ responses to each question.

## Results

### APIC Study (Phase 1)

During the training session conducted prior to the beginning of the data collection, 4 of the 5 data collectors were already familiar with the iPad and therefore needed to be instructed only on how to use the iSurvey app. We found that all expert raters, including 1 rater who was not previously familiar with the iPad, were easily and quickly familiarized and immediately able to use the iPad- and iSurvey-based data collection tool. Interrater agreement of the data collection tool was found to be very high for each of the three videotaped scenarios. Median Fleiss’ kappa for the three scenarios was 0.88 (95% CI, 0.78-1.00).

During the three meetings we conducted with the expert raters during the study, they did not report any problems and the data collection was completed without any problems. The expert raters and team members were able to answer the more than 60 questions of the data collection protocol in an average time of 25 minutes. The download of the data was convenient and fast, without any interruptions. We were able to export the data into MS Excel (Mac 2011).

For our 131 days of data collection, we needed to subscribe to iSurvey for 5 months. iSurvey is available from US $89 per month (December 2015). One subscription allows one survey to be completed up to 3000 times per month on an unlimited number of devices. Also, we acquired one iPad for each expert rater. The iPad is available from US $269 (iPad mini 2 WiFi, December 2015).

### Expert Survey (Phase 2)

The results of the expert survey contained Likert-type ratings and open-ended responses concerning multiple data collection aspects. The majority of raters were positive that the anesthesia team members (ie, the surveyed doctors and nurses) using the iPad- and iSurvey-based tool in the ORs liked it (see Table 1). Most clearly, the raters preferred the iPad- and iSurvey-based tool over a paper-and-pencil–based method they had used during other, earlier studies (median response 1, interquartile range [IQR] 1-1). Furthermore, they found the iPad (median 5, IQR 4.5-5) and iSurvey (median 4, IQR 4-5) worked flawlessly and was easy to use (median 5, IQR 4-5). Expert ratings also showed that the anesthesia team members (ie, the surveyed doctors and nurses) who used the iPad- and iSurvey-based tool in the ORs liked it (median 4, IQR 3-4.5). In addition, all observers provided answers to the open-ended question (question 7). Their unedited answers are given in Table 2.

**Table 1:** Survey of raters’ experience using iSurvey software on iPad tablet computers^a^.

Question	Rater 1	Rater 2	Rater 3	Rater 4	Rater 5	Median (IQR)
1. I found the iPad loaded with iSurvey easy to use for observing teams in the operating room.	5	5	5	4	4	5 (4-5)
2. The app iSurvey worked flawlessly during the study.	5	4	5	5	4	5 (4-5)
3. The iPad worked flawlessly during the study.	5	4	5	5	5	5 (4.5-5)
4. I would prefer to use paper and pencil over an iPad- and iSurvey-based tool when observing teams in the operating rooms.	1	1	1	1	1	1 (1-1)
5. The anesthesia team members liked using the iPad- and iSurvey-based tool to answer the questions in the operating rooms.	4	3	5	3	4	4 (3-4.5)
6. The anesthesia team members answering the questions had problems using the iPad- and iSurvey-based tool.	1	4	1	1	2	1 (1-3)
7. Were there any problems using the iPad in the operating rooms? Do you have any other remarks?	Open-ended question					

^a^1=do not agree, 2=somewhat disagree, 3=neutral, 4=somewhat agree, 5=fully agree.

**Table 2:** Unedited answers of the 5 observers to the open-ended question “Were there any problems using the iPad in the operating rooms? Do you have any other remarks?”

Number of observers	Unedited answer	Summary
1	No problems observed during the use of the iPad-based survey. Data can be better anonymized when using an iPad based survey, because as soon as the team members completed their survey the answers of the participant were not visible to us? (and did not have to be stored somewhere in the OR).	No problems; Data safety advantages
2	No real problems, though some hesitation by the team members could be noted, especially by means of hygiene and removal of gloves before use.	No serious problems; Uncertainties about hygiene
3	No problems, no remarks	No problems
4	During a certain period of time, the uploading didn’t work the way it should have, but in the end, everything was fine!	Problems with data upload
5	Having a tool of the size of a regular iPad is not always helpful in the busy routine of the OR (where to put it when finishing the survey…)	Remark about the size of the data collection tool

## Discussion

### Principal Findings

We conducted two studies that showed that the combination of iPad and iSurvey provided an efficient and effective way to collect observational and survey data in the OR. Based on the results of an intervention study that evaluated the use of an Anesthesia Pre-Induction Checklist (APIC), we found that the tool was suitable for both systematic observations of team performance during anesthesia inductions as well as team member surveys thereafter. Based on the results of a follow-up survey, we found that the iPad- and iSurvey-based tool was well accepted and easily used by the expert observers.

### Phase 1: Using the iPad and iSurvey to Observe Team Performance and Assess Team Member Perceptions

Comparing the results of the individual expert raters’ ratings of videotaped pre-induction scenarios allowed us to assess interrater reliability of the iPad- and iSurvey-based data collection tool. The high interrater reliability scores showed that (1) immediately after the rater training, expert raters were able to use the combined tool in the OR, and (2) the obtained data provided a reliable assessment of team performance during anesthesia inductions in the OR.

The training of the data collectors was effortless with the iPad- and iSurvey-based tool being immediately intuitive to the expert raters. The multi-touch finger-sensitive interface of the iPad is used in many Apple devices (eg, iPod, iPhone, Apple Watch), and many people are familiar with the use of this interface [20-22].

iSurvey integrates the name of the device from which data were entered in each rating record, for example, iPad #1. This made it easy to create subgroup analyses, for example, to compare the results of individual observers and to compute interrater agreement.

### Phase 2: Experiences of Expert Raters

The results of the expert rater survey showed that the majority of raters either completely or somewhat agreed that both the iPad hardware and iSurvey software worked flawlessly and they found it easy to use for observing teams in the OR. The data also revealed some additional experiences on the part of raters. For example, one rater stated that data collection is safer compared to a paper-and-pencil-based method, because unlike on paper, after the data has been entered it is not present on paper sheets lying around in the OR, where they could possibly be read by another person. Some minor problems were reported as well that are noteworthy to discuss. First, one rater stated that some study participants were hesitant to use the tool during the induction and remove their gloves before using the iPad. This is an important factor that comes into play when team members have to complete surveys immediately after an anesthesia induction. To facilitate ease-of-use in sterile environments, it might be useful to use a technique for sterile iPad use [5].

Another rater commented that the upload did not work during a certain time, which may have been caused by a temporary unavailability of iSurvey due to maintenance. This had been communicated to us in advance by Harvestyourdata and lasted for only a couple of hours. All data collected during this timeframe were saved on the iPads and could be uploaded once the service became available again.

Finally, 1 rater pointed out that there were some problems with bringing the iPad to the ORs and not knowing where to put it after an observation. This problem could be mitigated by using iSurvey on an iPhone, iPad mini, or iPod in future research projects. These devices are smaller in size but just as powerful.

In conclusion, this survey showed that the iPad- and iSurvey-based tool was well accepted and considered to work well by the sharp-end expert observers, who used the tool during an actual study. There were no major problems and the impressions of the expert raters were that the surveyed team members had neutral or positive sentiments towards the iPad- and iSurvey-based tool, but no negative sentiments.

### Theoretical and Practical Implications

The combined use of iPad and iSurvey enabled us to collect data seamlessly, which was especially important in the OR, which is an environment that allows only limited disturbance by researchers. Furthermore, the tool eliminated the need to transcribe data from paper sheets to electronic data files. The use of an electronic iPad- and iSurvey-based data collection tool may provide some important advantages over a pencil-and-paper–based approach. First, rater accuracy may be improved because there are no paper-based data collection forms to keep track of. Second, once the observations are completed, the dataset can be downloaded as a ready-to-use data file thus avoiding the risk of data transcription errors or errors in the assignment of papersheets to the correct dataset (likely to occur when paper-based observations need to be copied into electronic data files). Third, the number of datasets, which can be handled electronically, is practically unlimited. Furthermore, additional data points such as GPS position can be gathered by an electronic tool that might serve as a cross-check to the rater responses and thus further improve accuracy.

Our findings contribute to a better understanding of how multimethod field studies can be conducted and implemented in a context as sensitive as the OR. Prior research has focused on what kind of teamwork patterns should be observed in order to assess team performance in the OR [23], but few studies have outlined how to conduct such research. Our findings show that when teamwork episodes consist of short fragmented cycles (ie, during and after an anesthesia induction) and are distributed across multiple locations, a tablet computer such as the iPad combined with a ready-to-use survey app (iSurvey) is well suited to assess team performance and individual teamwork perceptions. We thus conclude that using the iPad combined with iSurvey may not only be a useful tool to assess data in the OR and other health care settings but in many other high-risk and action team settings such as aviation, mining, or the military. Given that our observers were also experienced clinicians who had to monitor the performance of the observed anesthesia team, the use of an iPad provided an effortless and very convenient way to carry and store the data collection tool at any time during the data assessment.

We also found that combining iPad and iSurvey can be used to observe as well as to survey study participants. Thus, using the iPad as a data collection tool can help triangulate different data sources to more accurately capture teamwork processes in the OR [24].

### Limitations

Notably, our study has some limitations, which we will outline below. First, it must be noted that we evaluated the use of the iPad- and iSurvey-based tool using parsimonious rating scales to assess team performance and team member perceptions in the OR. In order to use more sophisticated observation methods such as time-based or event-based rating methods (ie, counting and logging the frequency of a certain behavior occurring during a specified time frame), time-logging of a variety of behavioral markers during a team interaction period is necessary. For example, observation studies in the OR looking at team coordination or communication [25] have used coding schemes consisting of 12 or more behavioral codes. Using more complex coding schemes may surpass the capabilities of a ready-to-use survey software and require the use of a behavioral coding software. Future research may address how more complex behavioral coding manuals can be used in combination with tablet computers to facilitate real-time coding in the OR.

The results of this work are based on a single center study, and the expert rater survey featured a small sample size. Future work studying end user perceptions about electronic data collection tools should feature a larger sample size.

Like any data collection method, the use of the iPad- and iSurvey-based data collection tool must be evaluated and approved by the responsible ethics committee before the beginning of a research project. The use of an iPad- and iSurvey-based data collection tool, depending on the regulation in place, may not be possible in all countries for all research projects. One safety-critical limitation of an iPad- and iSurvey-based data collection tool is its use in an environment near a strong magnetic field, for example, a magnetic resonance imaging device. Electrical devices exhibit substantial magnetic field interactions such as translational attraction and heating. This could potentially injure a patient or a caregiver.

Further regulations that govern the use of computerized data collection protocols should be taken into account before conducting a study that involves the use of iPads in a hospital context. Consulting the Food and Drug Administration Code of Federal Regulations title 21 [26] and the European Medicines Agency guidelines for Good Clinical Practice may be important [27]. In all cases it should be ascertained that collecting data with iPad and iSurvey is in accordance with standards for human subject research as derived from the World Medical Association Declaration of Helsinki - Ethical Principles for Medical Research Involving Human Subjects [28].

### Conclusion

Based on the results of a large field study conducted in multiple operating room areas of a teaching hospital and a follow-up study of the expert raters who used the tool, we outlined and evaluated the combined use of iPad and iSurvey. We found that the use of the iPad- and iSurvey-based tool was suitable to observe teams in their natural environment, to collect clinical performance and communication data, and to survey team members immediately after they completed their task (ie, anesthesia induction). Additionally, it was positively evaluated by expert raters. Considering these comprehensive results, we can recommend the use of the iPad- and iSurvey-based tool for studying team performance and team member perceptions in the OR.

## Abbreviations

APIC: Anesthesia Pre-Induction Checklist
CFR: Code of Federal Regulations
IQR: interquartile range
KEK: Kantonale Ethik Kommission (Cantonal Ethics Commission)
OR: operating room
